# HDR Brachytherapy in the Treatment of Skin Kaposi Sarcoma: A Mono-Institutional Series

**DOI:** 10.3390/cancers18020319

**Published:** 2026-01-20

**Authors:** Bianca Santo, Elisa Ciurlia, Maria Cristina Barba, Elisa Cavalera, Rosa Coppola, Paola De Franco, Sara De Matteis, Giuseppe Di Paola, Angela Leone, Antonella Papaleo, Donatella Russo, Dino Rubini, Giuseppe Rubini, Angela Sardaro

**Affiliations:** 1 Radiation Oncology Unit, Department of Onco-Hematology, “Vito Fazzi” Hospital, 73100 Lecce, Italy; 2 UOS Dermatologia, Fondazione Policlinico Universitario Campus Bio-Medico, 00128 Roma, Italy; 3 Radiation Therapy Unit, Department of Precision Medicine, Università degli Studi della Campania Luigi Vanvitelli, 80129 Napoli, Italy; 4 Nuclear Medicine Unit, Interdisciplinary Department of Medicine, University of Bari, 70121 Bari, Italy

**Keywords:** Kaposi sarcoma, high-dose-rate contact brachytherapy (c-HDR-BRT), hypofractionated radiotherapy

## Abstract

**Background:** Kaposi sarcoma (KS) is a multifocal, angioproliferative neoplasm strongly associated with human herpesvirus-8 infection. Radiotherapy(RT) is a well established treatment due to the intrinsic radiosensitivity of KS lesions. High-dose-rate contact brachytherapy allows precise dose delivery with optimal sparing of surrounding tissues; however, its application in KS remains poorly documented. **Methods:** We conducted a retrospective analysis of 10 patients with histologically confirmed KS treated with c-HDR-BRT between June 2010 and June 2023. A total of 40 cutaneous lesions were treated using Leipzig applicators with hypofractionated regimens: 10 Gy in 1 fraction, 20 Gy in 2 fractions, or 30 Gy in 3 fractions. Treatment parameters were individualized based on lesion size and location. Local control (LC), overall survival (OS), disease-specific survival (DSS), and toxicity (graded by the RTOG criteria) were evaluated. Follow-up assessments were performed every four months during the first year and annually thereafter. **Results:** At a median follow-up of 10.3 years, the 2-year LC, OS, and DSS rates were 100%. Complete response was achieved in 62.5% of lesions, with a partial response observed in 37.5%. Grade 1–2 acute skin toxicities were recorded in 55% of treated lesions, while grade 3 toxicity occurred in a single case (2.5%) and was managed conservatively. The hypofractionated schedule significantly improved patient compliance, particularly in those with multiple lesions requiring sequential irradiation. **Conclusions:** Our long-term institutional experience supports c-HDR-BRT as a feasible and well tolerated local treatment option for the management of KS, providing favorable long-term local outcomes. These results support the inclusion of c-HDR-BRT in the multidisciplinary treatment of KS, warranting further prospective evaluation.

## Simple Summary

Kaposi sarcoma is highly radiosensitive, yet evidence on high-dose-rate contact brachytherapy (c-HDR-BRT) remains limited. In a retrospective series of 10 patients (40 lesions) treated with Leipzig applicators at our institution between 2010 and 2023, hypofractionated schedules of 10–30 Gy (1–3 fractions) achieved favorable long-term local outcomes. At a median follow-up of 10.3 years, 2-year local control, overall survival, and disease-specific survival were 100%. Complete response was observed in 62.5% of lesions, and partial response was observed in 37.5%. Treatment was well tolerated, with mostly grade 1–2 acute skin reactions and only one grade 3 event. The short treatment courses improved compliance, particularly for patients with multiple lesions. These findings support c-HDR-BRT as a feasible and well tolerated local treatment option for Kaposi sarcoma and justify further prospective evaluation.

## 1. Introduction

Kaposi sarcoma (KS) is a multifocal, angioproliferative neoplasm typically manifesting on the skin, but it can also affect internal organs. It is categorized as a low-grade vascular tumor, and it is linked to infection with Kaposi sarcoma herpesvirus/human herpesvirus 8 (KSHV/HHV8) [1]. This condition was initially documented by Moritz Kaposi in 1872 [2].

The development of KS takes place when the immune system is compromised, and it can manifest in four distinct clinical forms: (i) classic—a cutaneous presentation, frequently localized in the lower limbs; (ii) endemic—more prevalent in Africa, similar to the classic form but displaying heightened aggressiveness in younger patients; (iii) post-transplant—associated with immunosuppressive conditions following transplantation; and (vi) Human immunodeficiency virus (HIV)-related (epidemic KS)—linked to the HIV epidemic.

The clinical progression of KS demonstrates significant variability. Often, it pursues a benign course and, although progressively advancing, tends to remain localized to the skin and subcutaneous tissues, but it can affect all organs.

The evolution of KS lesions can be observed as early macules (patch stage) that transform into plaques (plaque stage), which subsequently expand into larger nodules (tumor stage). These lesions manifest as purplish, reddish-blue, or dark brown/black lesions, might bleed or ulcerate and can be accompanied by lymphedema, pain, and secondary infections [1].

A consensus regarding the most suitable tumor-directed therapy has not yet been reached. Management considerations for KS encompass factors such as the clinical variant, the extent and stage of tumor growth, immune system status, coexisting conditions, and symptomatology [3]. Several treatments have been documented, such as surgical excision, cryotherapy, laser ablation, photodynamic therapy, topical alitretinoin gel, and intralesional and systemic chemotherapy and radiation therapy [3].

The management of Kaposi sarcoma is primarily guided by disease extent and clinical behavior. In patients with localized disease (stage I–II), treatment is generally based on local approaches aimed at controlling symptoms and improving cosmetic outcomes. These include ablative techniques such as cryotherapy and laser therapy, topical agents, intralesional chemotherapy, and radiation-based treatments [4,5].

Radiotherapy, delivered either as external beam irradiation or contact brachytherapy, represents an effective option due to the high radiosensitivity of Kaposi sarcoma lesions, while surgical excision is usually limited to diagnostic purposes [6].

In contrast, patients with progressive or metastatic disease (stage III–IV) typically require systemic treatment. Liposomal doxorubicin is commonly used as first-line chemotherapy, with paclitaxel as an alternative option. Additional systemic strategies include antiangiogenic agents and immunotherapeutic approaches, as well as other immunomodulatory or targeted therapies currently under clinical investigation. In selected cases, radiotherapy may still play a complementary role within the management of symptomatic or visceral lesions when systemic therapy is contraindicated or insufficient.

From a radiobiological standpoint, the high radiosensitivity of Kaposi sarcoma is related to both the proliferative activity of spindle cells and the susceptibility of tumor-associated endothelial cells to radiation-induced apoptosis. This dual mechanism explains the rapid clinical regression frequently observed after relatively low radiation doses [7].

Given the high radiosensitivity of KS lesions, a common treatment approach for achieving remarkable local control is radiotherapy. Considering that the disease typically manifests as solitary lesions, the prevalent strategies often involve localized irradiation using low-energy electrons or superficial X-rays.

As a result, RT is now a crucial component of KS treatment. RT can be utilized as palliative therapy in the disseminated forms, as the primary treatment, and in cases of isolated lesions. Both cutaneous and mucosal lesions respond successfully to radiotherapy treatment. The NCCN recommendation [8] states that radiation is recommended when a cutaneous limited lesion is bothersome and/or unattractive. RT is a successful treatment for decreasing edema, halting bleeding, and relieving pain. In cases of chronic illness, RT is highly well tolerated and has the potential to enhance quality of life, lesion appearance, and symptoms.

The benefits of employing high-dose-rate brachytherapy (HDR-B) for KS treatment in contrast to the utilization of external beam radiation therapy (EBRT) [9] encompass superior dose distribution and enhanced preservation of subcutaneous tissues adjacent to the tumor.

Iridium-192 (Ir-192) is the radionuclide that HDR-BRT uses to make contact. A tungsten applicator with a specifically designed cup form is used, and the radiation source is positioned in close proximity to the tumor during contact HDR-BRT. Because Ir-192 generates gamma radiation with an energy level of 360 keV, it can be used as a source for brachytherapy to treat tiny, superficial skin cancers that are less than 5 mm thick [10].

In this study, we report our monoinstitutional experience at the Radiotherapy Unit of “Vito Fazzi” Hospital in Lecce, Italy, regarding the treatment of KS with hypofractionated c-HDR-BRT, with emphases on efficacy and safety outcomes.

## 2. Materials and Methods

The hypofractionated approach was deliberately chosen to optimize patient compliance, particularly in subjects presenting with multiple synchronous or metachronous lesions requiring sequential irradiation over time [11].

This analysis includes patients with KS treated with high-dose-rate contact brachytherapy (c-HDR-BRT) at the Radiation Brachytherapy Unit of the “Vito Fazzi” Hospital (Lecce, Italy) between June 2010 and June 2023. All patients were evaluated by a multidisciplinary group that included dermatologists, plastic surgeons, oncologists, radiation oncologists, and pathologists. The characteristics of the patients and lesions are summarised in Table 1. The histopathological confirmation of KS was obtained for all patients included in the study.

**Table 1:** Patient and lesion characteristics.

Age (Years)	72 (38–80)
**Gender**	Number (%)
Male	100
Female	0
**Lesion sites**	**% (n lesions)**
Upper limb	30 (12)
Lower limb	17.5 (7)
Hands/Feet	10 (4)
Trunk	42.5 (17)

During the multidisciplinary board examination, gross tumor volume (GTV) was identified; a margin of 0.5–1 cm was added to define the clinical target volume (CTV), depending on the size of the lesion:

- lesion < 2 cm: margin 0.5 cm;
- lesion ≥ 2 cm: margin 1 cm.

Depending on the extension of the CTV on the skin, Leipzig applicators of 10 mm (H1), 20 mm (H2), or 30 mm (H3) were utilized.

The prescription doses were as follows:

- 10 Gy in 1 fraction;
- 20 Gy in 2 fractions, delivered two times a week;
- 30 Gy in 3 fractions, delivered three times a week, with a minimum interval of 48 h between fractions.

Different doses have been used in relation to the anatomical site of the lesions.

During each treatment session, the patient was immobilized using an adjustable arm and adhesive material. The dose prescription point was located 3 mm to 5 mm below the skin surface.

Acute and late toxicities were assessed using the Radiation Therapy Oncology Group (RTOG) scale. Local control (LC), overall survival (OS) and disease-specific survival (DSS) were calculated using the Kaplan–Meier method. For the first year, follow-up appointments were set up every four months. A reduction of the palpable tumor to less than 50% of the pre-treatment area was considered a partial response, whereas a complete response was defined as the palpable tumor being completely resolved.

Any type of recurrence was considered skin advancement; progressions were evaluated both at the treated sites and at other sites.

Response evaluation was performed at both lesion and patient levels. Local response (complete or partial response) and toxicity were assessed on a per-lesion basis, as each cutaneous lesion represented an independent treatment volume with potentially different size, location, and dosimetric parameters. Conversely, overall survival (OS) and disease-specific survival (DSS) were analyzed on a per-patient basis, reflecting systemic disease behavior and patient-level outcomes. This dual-level analysis was intentionally adopted to accurately capture the local efficacy and safety of c-HDR-BRT while preserving the clinical relevance of survival endpoints.

## 3. Results

In the study, we evaluated 10 patients (all men) with a median age of 72 years (range: 38 years–80 years), and we treated a total of 40 lesions The patients were treated with c-HDR-BRT between June 2010 and June 2023. Histopathological examination of the skin was performed in all patients with a diagnosis of KS. Four patients (40%) were HIV-positive. Four patients (40%) had classic KS, one (10%) had the endemic subtype, and one (10%) had iatrogenic KS. All patients presented with exclusively cutaneous disease.

All patients were candidates for c-HDR-BRT due to the size of lesions.

The 40 lesions were located as follows: 12 at the level of the upper limb (30%), 7 at the level of the lower limb (17.5%), 17 at the level of the trunk (42.5%), and 4 at the level of hands and feet (10%).

Four lesions (10%) received 10 Gy in a single fraction, three (7.5%) received 30 Gy in 3 fractions, and 33 (82.5%) received 20 Gy in 2 fractions. The applicator size was H1 in 7 lesions (17.5%), H2 in 20 lesions (50%), and H3 in 13 lesions (32.5%).

The mean treatment delivery time was 762 s.

Three different doses and fractionation regimens were adopted: 30 Gy in three fractions, 20 Gy in two fractions, or 10 Gy in 1 fraction. The choice of fractionation (10 Gy/fraction) is aimed at concentrating the treatment to ensure patient compliance, as they often have multiple lesions to irradiate sequentially.

The mean follow-up period was 10.3 years (range: 1.8 months–12.2 years).

The 2-year local control (LC), overall survival (OS), and disease-specific survival (DSS) rates were 100%. A 100% local control rate was achieved, as no progression within the treated field was recorded. Nevertheless, disease progression at sites outside the radiation field was observed (as explained by the fact that 40 lesions were irradiated in 10 patients).

All patients used eudermic cream.

A patient undergoing HDR-BRT on 5 lesions died from another cause.

Acute toxicity was recorded in 22 lesions treated (55%):

- Grade 1 erythema appeared in fifteen cases with late persistence in five cases.
- Grade 2 erythema appeared in six cases: regression to grade 1 in two cases and progression to super intensive ulcer from Pseudomonas Aeruginosa in one case, treated by specialist.
- Towards the end of each treatment schedule, epidermolysis developed, which was resolved within 3 weeks.
- Grade 3 skin toxicity appeared in one case (2.5%) and was managed with dressings and close outpatient follow-up until grade 1 toxicity was achieved.

Treatments and toxicity are summarised in Table 2.

**Figure 1:**
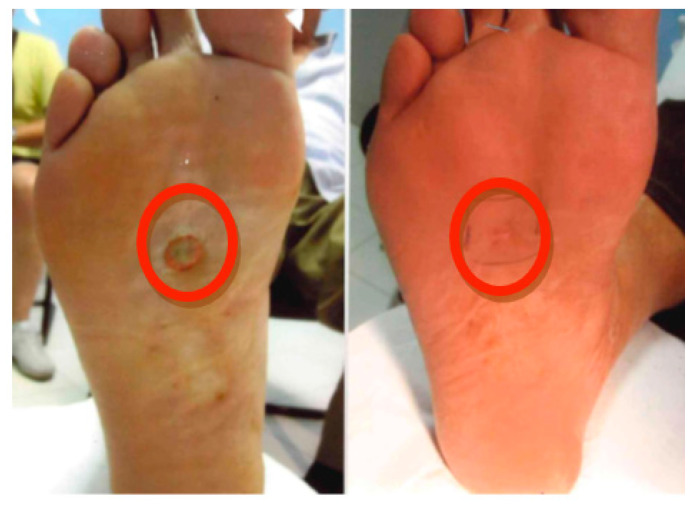
Lesion treated with brachytherapy at baseline and 6 months after treatment.

**Figure 2:**
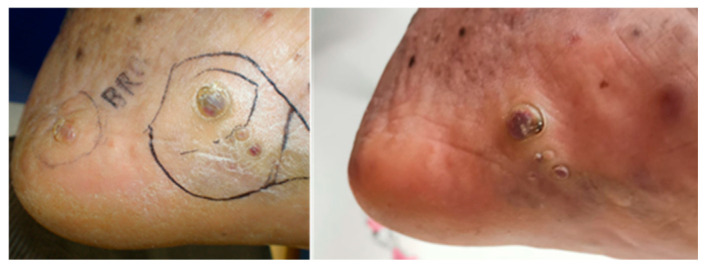
Lesion treated with brachytherapy at baseline and 6 months after treatment.

At the last follow-up, 62.5% (25/40 lesions) of patients were disease-free and 37.5% (15/40) recorded a partial response (defined as “palpable tumor < 50%”). Patients underwent dermatological and radiotherapy follow-up for the first two years every six months and subsequently annually if the response persists. Photographic images were collected; however, they are not standardized for all patients, partly due to the time intervals between data collection. We have reported two clinical cases treated with HDR-BRT in Figure 1 and Figure 2.

**Table 2:** Treatment characteristics.

**Dose and Fractionation**	
10 Gy/1 fx	
20 Gy/2 fx	
30 Gy/3 fx	
Leipzig applicator size *	
H1 17.5%	
H2 50%	
H3 32.5%	
Treatment delivery time sec 762	
Doses and Fractions *	
30 Gy/3 fx	7.5%
20 Gy/2 fx	82.5%
10 Gy/1fx	10%
RTOG Acute Toxicity *#	
G0 45%	
G1 37.5%	
G2 15%	
G3 2.5%	
RTOG Late Toxicity *#	
G0 70%	
G1 12.5%	
G2 15%	
G3 2.5%	

* The percentages are calculated based on the number of lesions treated. # All toxicities are reported per lesion and refer to the maximum grade observed per lesion.

## 4. Discussion

The absence of in-field recurrences in our series supports the hypothesis that adequate local dosing with c-HDR-BRT is sufficient to achieve durable tumor control. New lesions developing outside the treated field should be interpreted as disease progression rather than treatment failure, reflecting the systemic nature of Kaposi sarcoma [7,11]. The interpretation of our results should take into account the lesion-based nature of local response and toxicity analyses. While per-lesion evaluation is commonly adopted in studies involving multifocal cutaneous diseases such as Kaposi sarcoma, this approach may overestimate treatment efficacy in patients presenting with multiple lesions. However, in the context of contact brachytherapy, each lesion constitutes a distinct target with individualized dosimetric characteristics, justifying lesion-level assessment for local control and toxicity. Importantly, patient-based endpoints, including overall survival and disease-specific survival, were analyzed at the patient level to avoid misinterpretation of systemic outcomes. Nevertheless, the lesion-based analysis represents a methodological limitation of this study and should be considered when comparing results across different treatment series.

The multifocal and often recurrent nature of KS poses a significant therapeutic challenge. In this context, treatment modalities that combine high efficacy with minimal toxicity and logistical simplicity are particularly valuable. Hypofractionated c-HDR-BRT allows treatment completion within a limited number of sessions, reducing hospital visits and improving adherence, especially in elderly patients or those with comorbidities.

It is generally agreed upon in the literature that local disease, especially maculonodular lesions, can be controlled by radiotherapy. KS’s high radiosensitivity has been recognized since 1900 [12]; radiotherapy has thus become a primary treatment for localized forms and a palliative approach for disseminated forms, with local symptoms such as bleeding or pain being effectively reduced.

Kasper et al. demonstrate that HDR brachytherapy treatment is an effective, non-invasive option for patients with small cutaneous Kaposi sarcoma lesions. Cosmetic results appear excellent for most patients, and fewer treatment sessions over a shorter period offer greater convenience and less disruption to daily routines than other common regimens consisting of 2–3 weeks of daily treatments [13]. An additional advantage of contact brachytherapy lies in the excellent cosmetic outcomes. Preservation of surrounding healthy tissue is crucial for lesions located in visible or functionally sensitive areas such as the hands, feet, and face. In our series, no relevant cosmetic impairment was reported, supporting the esthetic safety of this approach.

Treatment of KS with external beam brachytherapy is supported by several studies [14,15], and high-dose cutaneous brachytherapy seems to be an effective strategy in the treatment of KS lesions [16,17]; even if Leipzig applicators are widely used for HDR-BRT for NMSC (non-melanoma skin cancer) [18,19,20], the same is not true for KS.

As already established [21], due to the limitations of the linear–quadratic model, which is not applicable for doses higher than 7 Gy–8 Gy per fraction [21,22], it would be incorrect to make a dosimetric comparison between this study and other series of studies in terms of the calculated Biological Effective Doses (BED) and Equivalent Dose (EQD2) (setting the alpha/beta ratio for skin cancer at 10).

It is well known that KS has a high level of radiosensitivity. There is currently no consensus on the standard treatment, and there is no precise definition of the optimal radiation dose to provide.

Since most studies have employed doses between 20 Gy and 30 Gy, KS is responsive to moderate radiation exposures. According to previously published research, the overall tumor response rate ranged from 47% to 99% [13]. The radiosensitivity of KS is the same for all types: endemic (as found in Africa), epidemic (AIDS-associated), iatrogenic (connected to immunosuppressive medication), and classical or sporadic.

Complete response rates of 98.7% and 91.43%, respectively, were noted in retrospective research conducted by Caccialanza et al. on 711 lesions of typical KS and 771 lesions of HIV-related KS that were treated with radiation [21].

A recent meta-analysis [18] examined the studies in the literature regarding the use of EBRT in Kaposi sarcoma, highlighting significant heterogeneity in the doses and fractionations employed. High-dose brachytherapy has proven to be very effective [19]. In fact, 20–30 Gy administered over 2–3 weeks has yielded excellent results [16,17].

EBRT continues to play a central role in its management despite major advances in systemic therapies and antiretroviral treatment [23,24].

One of the most consistent findings across published series is the high local control rate achieved with EBRT in Kaposi sarcoma. Response rates exceeding 80–90% have been reported regardless of disease subtype, lesion location, or fractionation schedule. This remarkable radiosensitivity likely reflects both the intrinsic vulnerability of proliferating spindle cells and the susceptibility of the abnormal tumor vasculature to radiation-induced damage. From a clinical perspective, these biological features translate into rapid tumor regression and prompt symptom relief, which are particularly valuable in patients with painful, bleeding, or function-limiting lesions. In the era of combination antiretroviral therapy (ART), the epidemiology and clinical behavior of HIV-associated KS have changed substantially. While systemic control has improved, localized symptomatic disease remains common, especially during immune reconstitution or in patients with partial virologic suppression. In this context, EBRT serves as an effective adjunct to systemic management, offering local disease control without interfering with ART. Another key aspect emerging from the literature is the flexibility of EBRT dose and fractionation schedules. Both conventional fractionation (20–30 Gy in 10–15 fractions) and hypofractionated regimens (e.g., 8 Gy in a single fraction or 16 Gy in 4 fractions) have demonstrated comparable efficacy in terms of tumor response and symptom palliation.

Toxicity associated with EBRT is generally mild and manageable. Acute effects include erythema, desquamation, and hyperpigmentation, while late toxicities are uncommon due to the relatively low doses employed [25]. Severe complications are rare even in immunocompromised patients [8]. Despite its established efficacy, EBRT does not address the systemic nature of Kaposi sarcoma. Local recurrences or the appearance of new lesions outside the treated field may occur, emphasizing the importance of integrating radiotherapy into a broader therapeutic strategy [26].

In conclusion, EBRT remains a cornerstone in the management of Kaposi sarcoma. Its high efficacy, favorable toxicity profile, and adaptability to different clinical scenarios ensure its continued relevance in modern oncology practice. To our knowledge, there are only two studies that exclusively analyze KS treated with contact brachytherapy, unlike what happens in non-melanoma skin cancer.

Kasper et al. have published the only report on a KS skin lesion treated with HDR brachytherapy. They analyzed sixteen patients treated in four to six fractions with the Leipzig applicator and with doses varying from 24 to 35 Gy (EQD2 28–43.75 Gy), recording a complete response [13].

A second study evaluated the use of Valencia applicators, which include a flattening filter to improve dose distribution by administering 25 Gy in 5 fractions (EQD2 31.5 Gy), two to three times a week. In this case as well, a complete response was recorded in all cases [9]. An optimal efficacy and safety profile was therefore documented with a limited treatment duration, which allowed greater patient compliance.

There are various experiences for different pathologies that make use of contact brachytherapy. Table 3 summarises studies of HDR brachytherapy with contact applicators in KS.

A study describes a single-center experience with HDR-BT for the treatment of mycosis fungoides involving anatomically complex and highly curved skin sites. In 28 patients with 39 treated lesions, HDR-BT achieved excellent dosimetric conformity and a 100% complete local response rate, including in a substantial proportion of previously irradiated areas. Treatment-related toxicity was low and predominantly mild, supporting the favorable safety profile of this technique. These findings suggest that HDR-BT is an effective alternative to conventional radiotherapy modalities for cutaneous lesions in challenging locations, allowing adequate target coverage with rapid dose fall-off and reduced exposure of underlying organs at risk [27].

A systematic review evaluates the role of brachytherapy in the management of primary and recurrent vulvar cancer, highlighting its potential as a conservative and organ-sparing treatment option. Across nine retrospective studies including 177 patients, brachytherapy—used alone or in combination with external beam radiotherapy—was associated with encouraging long-term outcomes in both primary and recurrent settings, with acceptable rates of local control, disease-free survival, and overall survival. Importantly, treatment-related toxicity was generally low, with infrequently reported severe late adverse events despite the frequent use of re-irradiation. Although the available evidence is limited by heterogeneity in techniques, doses, and study design, these findings support brachytherapy as a feasible and effective option for selected patients, particularly those unfit for surgery or with recurrent disease, when delivered in experienced multidisciplinary centers [28].

A cohort study evaluates the clinical effectiveness, toxicity, cosmetic outcomes, and patient-reported outcome measures of high-dose-rate mold-based brachytherapy for cutaneous neoplasms in an elderly and predominantly frail population. In 64 patients, most of whom presented with facial lesions and were unsuitable for surgery or definitive external beam radiotherapy, mold-based HDR brachytherapy achieved high rates of durable local control with a low incidence of severe late toxicity. Despite the anatomical complexity of treated sites, treatment was generally well tolerated, and cosmetic outcomes were rated as good or excellent by the majority of patients. Importantly, the inclusion of patient-reported outcomes highlights the acceptable treatment burden and quality-of-life impact of this approach. These findings support mold-based HDR brachytherapy, including the use of individualized and three-dimensional-printed applicators, as a safe and effective treatment option for selected patients with cutaneous neoplasms, particularly in cosmetically sensitive areas and in those unfit for standard curative therapies [29].

Although EBRT remains widely used, it may be associated with larger irradiated volumes and longer treatment courses. Conversely, c-HDR-BRT offers steep dose gradients, limiting exposure to adjacent structures and making it particularly suitable for superficial lesions smaller than 5 mm in thickness.

**Table 3:** Summary of studies on HDR brachytherapy with contact applicators in KS.

Author	Year	Histology	N. Lesions N. Patients	Macroscopic Lesion/Microscopic Margins	Applicator	N. fx	Dose/fx	Frequency	Total Dose	Follow-Up (Median)	Local Control	Acute Toxicity	Late Toxicity
Köhler-Brock [30]	1999	BCC SCC Kaposi’s sarcoma Lymphomas Melanomas	520 lesions 520 patients	NA	Leipzig	4–8	5–10 Gy	1–2 times a week	30–40 Gy	10 years	91%	NA	NA
Delishaj [31]	2015	BCC (44) SCC (12) Kaposi’s Sarcoma (1)	57 lesions 39 patients	45 macroscopic 12 positive margins	Valencia	8–10	5 Gy	2–3 times a week	40–50 Gy	12 months	96%	G1 58% G2 5.3%	G1 17% G2 1.9%
Kasper [13]	2013	Kaposi’s Sarcoma	16 lesions 16 patients	16 macroscopic	Leipzig	4–6	6-7 Gy	2–3 times a week	24–35 Gy	41.4 months	100%	G1 81.3% G2 6.3%	G2 6.3%
Ruiz [2]	2017	Kaposi’s Sarcoma	5 lesions 3 patients	5 macroscopic	Valencia	5	5 Gy	2–3 times a week	25 Gy	15 months	100%	G2 20%	0

## 5. Conclusions

Overall, these findings support the role of c-HDR-BRT as an effective and well tolerated therapeutic modality for the treatment of KS, providing robust oncological control while maintaining a favorable safety profile. The limitations of this study include its retrospective design and the relatively small number of patients. However, the extended follow-up period represents a major strength, allowing for the assessment of long-term disease control and late toxicity, which are often underreported in KS series. Importantly, this technique appears to mitigate treatment-related functional limitations and cosmetic alterations—factors that are particularly relevant given the often multifocal and visible nature of KS lesions. From a clinical standpoint, the absence of in-field recurrences observed in our cohort reinforces the concept that local control of KS lesions is achievable with appropriately selected radiation doses. Importantly, new lesions occurring outside the treated field reflect the systemic nature of the disease rather than local treatment failure. The results further highlight the necessity of tailoring therapeutic decisions to the individual patient, taking into account disease extent, lesion characteristics, and patient-specific clinical factors. Such a personalized approach is essential to optimize treatment outcomes, enhance quality of life, and achieve the best possible balance between therapeutic efficacy and toxicity in the management of KS. Future research should focus on prospective multicenter studies to better define optimal dose-fractionation schedules and to directly compare c-HDR-BRT with EBRT in terms of efficacy, toxicity, patient-reported outcomes, and cost-effectiveness. Integration of quality-of-life metrics would further enhance the clinical relevance of such investigations.

## Data Availability

The original contributions presented in this study are included in the article. Further inquiries can be directed to the corresponding author.
